# A pulmonary hamartoma in Covid-19 pneumonia: an interesting case studied with computed tomography

**DOI:** 10.1016/j.radcr.2021.01.051

**Published:** 2021-01-31

**Authors:** Francesco Messina, Grazia Calabrese, Lorena Turano, Carmela Tebala, Nicola Arcadi

**Affiliations:** Unit of Radiology, Riuniti Hospital, Azienda Ospedaliera Grande Ospedale Metropolitano (G.O.M.) “Bianchi-Melacrino-Morelli”, Via Giuseppe Melacrino n.21, 89124 Reggio Calabria, Italy

**Keywords:** Pulmonary hamartoma, Calcifications, Covid-19, Pneumonia, Computed tomography

## Abstract

Since the widespread of acute respiratory syndrome infection caused by Coronavirus-19, chest computed tomography (CT) was considered a useful imaging tool commonly used in early diagnosis and monitoring of patients with complicated Covid-19 pneumonia. Many typical imaging features of this disease were carefully described with chest CT, as well as the collateral CT findings in the lungs and mediastinum. Here we describe the case of a patient with Covid-19 pneumonia, that collaterally had a pulmonary hamartoma in the left lung, documented at CT.

## Background

Since December 2019 the world is facing a rapidly expanding pandemic of lower respiratory tract infection by a novel coronavirus SARS-CoV-2 (severe respiratory syndrome coronavirus-2). In some patients, this viral infection causes a clinical syndrome referred to as coronavirus disease 2019 (Covid-19), but the heterogeneity of the disease course poses a challenge to healthcare providers and optimal management of patients. The use of CT imaging in the diagnosis and follow-up had rapidly grown, and radiological patterns along the disease course are increasingly understood. Current guidelines advocate the use of noncontrast chest CT for the diagnosis, severity assessment, and monitoring of Covid-19 disease [1]. Chest CT imaging has been demonstrated more sensitive than chest radiography to identify the manifestations of Covid-19 pneumonia [2].

## Case presentation

A 62 years old man presented at emergency department with a history of ill-defined left sided chest discomfort since about ten days, with dry cough, shortness of breath and fever (37.3°C). The patient had been treated at home with paracetamol, without any improvement in his symptoms. He reported that he had a work-contact (about 3 weeks ago) with a positive Covid-19 man. Chest clinical auscultation revealed a bi-basal reduced intensity of breath sounds (more on the left). Arterial oxygen saturation (SaO2) was 90%. Laboratory exams showed increased values of C-reactive protein and procalcitonin, with leukocytosis.

The nasopharyngeal sampling was positive for SARS-CoV-2.

**Fig. 1:**
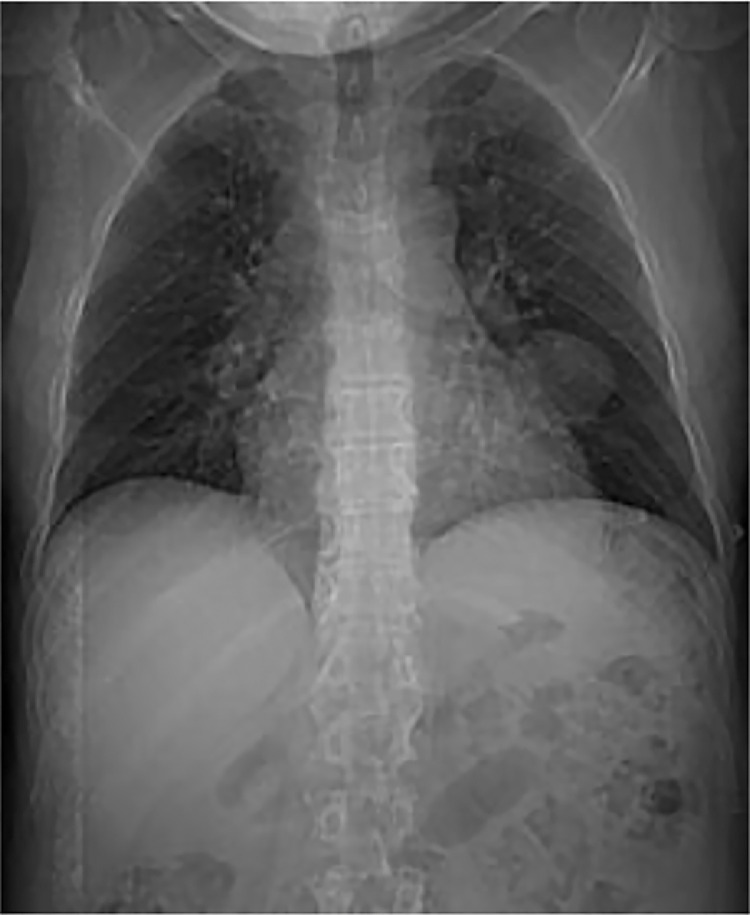
Chest X-ray showed accentuation of the peribroncovasal texture, and reticular thickening. It also showed a large mass lesion on the left.

A chest X-ray (only supine decubitus) was immediately made (Fig. 1). It showed an accentuation of the peribroncovasal texture, with fine reticular thickening. But it also showed a large mass lesion in the left mid-lower lung field.

**Fig. 2:**
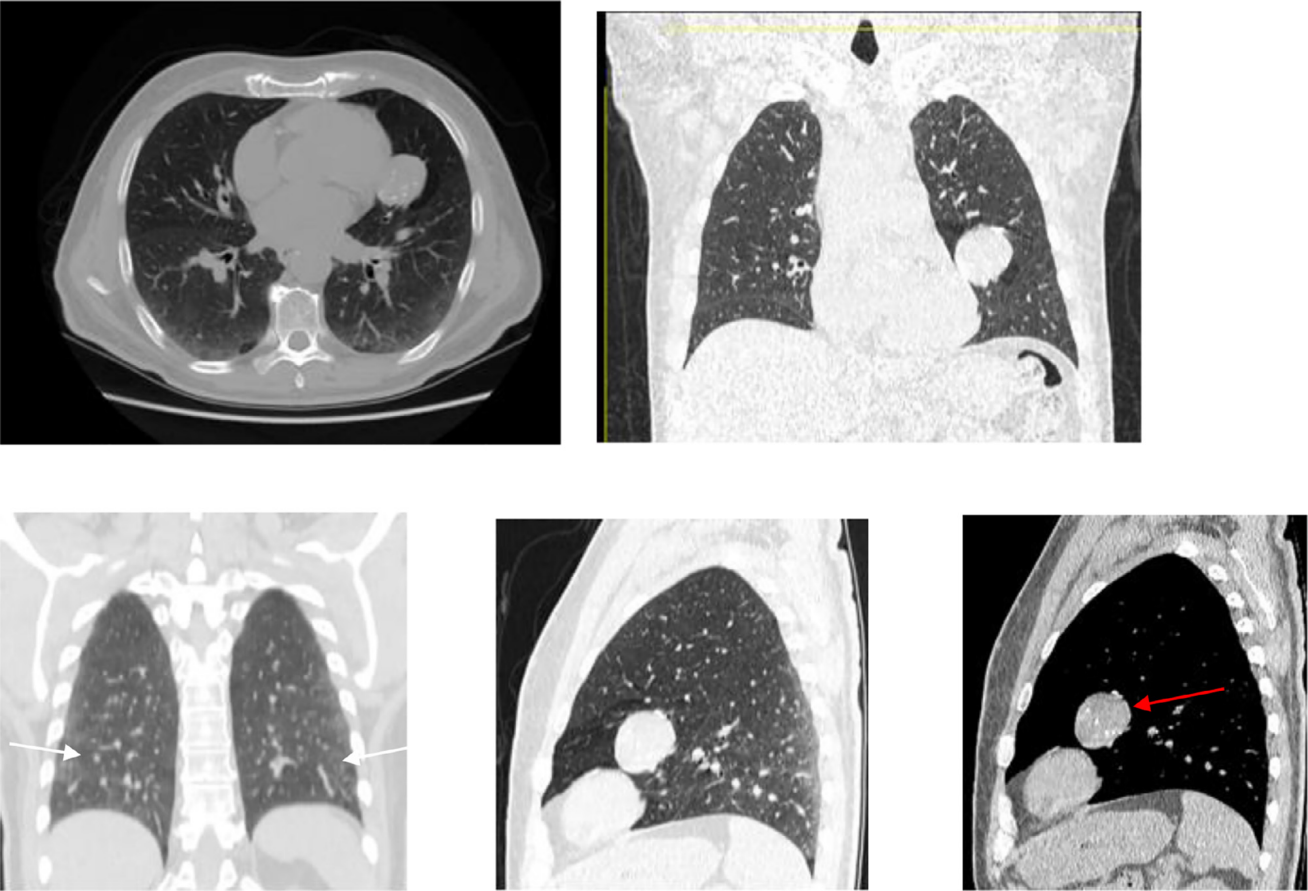
(a–e): CT showed in the basal regions of both lungs the presence of initial areas with “ground glass” pattern, with interstitial thickenings, as initial Covid-19 bilateral pneumonia (white arrows). CT also demonstrated a solitary, large (4 × 4 cm) mass lesion, in the left lung, with numerous and minute intralesional calcifications (“popcorn” calcifications; red arrow). (Color version of figure is available online.)

A chest CT (Figs. 2a–e) scan was urgently performed, in basal conditions, with a 64-slices multidetector scanner, and the images so obtained were analyzed with a slice-thickness of 1.2 mm and MPR reconstructions (axial, sagittal, and coronal). CT had documented in the basal regions of both lungs the presence of initial areas with “ground glass” pattern, with interstitial thickenings, as initial Covid-19 bilateral pneumonia. But CT also demonstrated a solitary, large (4 × 4 cms) mass lesion, in the left lower lobe, in the paramediastinal area and parascissural, with numerous and minute intralesional calcifications. The margins of the mass did not show spiculations. There was no evidence of mediastinal or hilar lymphadenopathies, neither other nodules in both lungs, nor any evidence of pleural and pericardial effusions. The pattern of calcification resembled a little “popcorn.” In view of characteristic radiological features, a diagnosis of pulmonary hamartoma (in Covid-19 bilateral pneumonia) was considered.

A CT-guided fine needle aspiration cytology from the mass was performed, and showed predominantly chondromyxoid matrix without any bronchial epithelium, neither necrosis or granulomatous inflammation. So, the diagnosis of a benign pulmonary hamartoma was confirmed.

The patient was treated, for the Covid-19 pneumonia, with drug therapy at home (fiduciary home isolation), with a progressive improvement of his general clinical conditions. After 10 days, the nasopharyngeal sampling had become negative for SARS-CoV-2. Pulmonary hamartoma was decided to follow-up over time, with CT imaging.

## Discussion

In the literature, several studies are reporting parenchymal findings of Covid-19 pneumonia. CT findings of Covid-19 pulmonary infection are: bilateral, peripheral, and basal predominant GGO; crazy-paving pattern; consolidations; nodules; reticulations; interlobular septal thickenings; linear opacities; subpleural curvilinear lines; bronchial wall thickenings, often with an extensive geographical distribution. Unenhanced chest CT is useful in early diagnosis of Covid-19 infection, in monitoring disease progression, coinfection, or disease stability. Chest CT can accurately evaluate the type and extent of lung lesions [3], [4], [5], [6]. Pulmonary hamartomas, accounting for approximately 6% of all solitary pulmonary nodules, are the most common benign lung tumors and are usually asymptomatic. They can rarely cause symptoms due to large size. Most are parenchymal, but uncommonly can be present in an endobronchial location. They are more common in males with peak incidence in the sixth or seventh decade of life [7]. The presence of “popcorn” calcification in a pulmonary hamartoma is one such characteristic radiologic “food sign.” The typical radiological pattern of popcorn calcification (seen in 10%-15% of pulmonary hamartomas), when present, is highly suggestive of the diagnosis [8,9]. Asymptomatic hamartomas usually require no further treatment. The indications of removal include a rapid growth of tumor with symptoms, suspected primary or secondary malignant tumor or endobronchial location of lesion with postobstructive complications. Surgical removal is the curative modality in such instances [10].

## Conclusions

CT plays an important role in the diagnosis and severity evaluation of Covid-19 pneumonia, because it investigates very well the dynamic CT changes in different stages of the disease, and also in the follow-up of the patients. CT scan has been shown to be very important in detecting calcifications of the hamartomas. Wide use of CT in the future should lead to a higher frequency of rilevation of calcifications in pulmonary hamartomas.

## Patient consent statement

The patient confirmed the consense for publication of our case report.
